# Causal assessment in evidence synthesis: A methodological review of reviews

**DOI:** 10.1002/jrsm.1569

**Published:** 2022-06-09

**Authors:** Michal Shimonovich, Anna Pearce, Hilary Thomson, Srinivasa Vittal Katikireddi

**Affiliations:** ^1^ MRC/CSO Social & Public Health Sciences Unit University of Glasgow Glasgow UK

**Keywords:** causal assessment, causality, population health, systematic review

## Abstract

In fields (such as population health) where randomised trials are often lacking, systematic reviews (SRs) can harness diversity in study design, settings and populations to assess the evidence for a putative causal relationship. SRs may incorporate causal assessment approaches (CAAs), sometimes called ‘causal reviews’, but there is currently no consensus on how these should be conducted. We conducted a methodological review of self‐identifying ‘causal reviews’ within the field of population health to establish: (1) which CAAs are used; (2) differences in how CAAs are implemented; (3) how methods were modified to incorporate causal assessment in SRs. Three databases were searched and two independent reviewers selected reviews for inclusion. Data were extracted using a standardised form and summarised using tabulation and narratively. Fifty‐three reviews incorporated CAAs: 46/53 applied Bradford Hill (BH) viewpoints/criteria, with the remainder taking alternative approaches: Medical Research Council guidance on natural experiments (2/53, 3.8%); realist reviews (2/53, 3.8%); horizontal SRs (1/53, 1.9%); ‘sign test’ of causal mechanisms (1/53, 1.9%); and a causal cascade model (1/53, 1.9%). Though most SRs incorporated BH, there was variation in application and transparency. There was considerable overlap across the CAAs, with a trade‐off between breadth (BH viewpoints considered a greater range of causal characteristics) and depth (many alternative CAAs focused on one viewpoint). Improved transparency in the implementation of CAA in SRs in needed to ensure their validity and allow robust assessments of causality within evidence synthesis.

**Highlights**

**What is already known**

Despite the potential benefits, there is currently no comprehensive and agreed upon approach for incorporating causal assessment approaches (CAAs) in systematic reviews (SRs) and reviews of reviews (RoRs).

**What is new**

To our knowledge this is the first methodological review to establish current practice of CAAs in SRs. Bradford Hill viewpoints (sometimes called criteria) were the most commonly used, but how they were implemented and transparency in reporting implementation varied greatly. There was overlap across the approaches with some focusing on one or two viewpoints while others considering several elements of causal assessment.

**Potential impact for RSM readers outside the authors' field**

For CAAs to be incorporated into SRs/RoRs across all fields, investigators must ensure transparency in choice of viewpoints and clarity around implementation, including justification or guidance used to inform operationalisation.

This methodological review offers examples of how CAAs can be implemented to maintain the transparency, robustness, and rigorous approach of SRs.

## 1 INTRODUCTION

Causal assessment involves researchers and policy makers interrogating the evidence to understand if a cause‐and‐effect relationship exists between an exposure and an outcome.^1^, ^2^ By bringing together evidence surrounding a research question, evidence synthesis is arguably preferable to relying on an individual study for causal assessment.^3^ This is particularly true in population health where evidence is mixed and potential causes are complex.^4^

The utility of evidence synthesis, including systematic reviews (SRs), in causal inference depends both on review conduct (which should be done as rigorously and transparently as possible^5^) as well as what evidence is synthesised. The types of studies included in SRs may affect the certainty of a causal relationship. This may be especially important where the available evidence is predominantly from non‐randomised studies (NRSs)^4^, ^6^ where there is a high risk of bias due to confounding when compared to randomised controlled trials (RCTs),^7^, ^8^ as is common in SRs addressing population health questions.^4^ Results from NRSs, even those with large sample sizes,^9^ may be misleading if not interpreted in light of potential sources of bias^10^ and may threaten the potential for SRs to evaluate causality.

The approach to evidence synthesis to evaluate a putative causal link between an exposure and outcome may differ from evaluating an association between an exposure and outcome.^5^, ^8^ To improve the assessment of causality, methods used in SRs may need to be adapted.^11^ While there are not clearly defined and agreed means of adjudicating causality, including within SRs,^11^ there are various guidelines and approaches that can be used to assess one or more aspects of causality.^4^ Going forward, the guidelines and approaches used to assess causality will be referred to as causal assessment approaches (CAAs), with the Bradford Hill (BH) viewpoints or criteria particularly influential. They may be incorporated into the evidence synthesis—sometimes referred to as ‘causal reviews’—to help establish if a causal relationship exists.^11^

Some CAAs, such as the BH, qualitatively evaluate different characteristics of causal relationships.^3^ BH viewpoints address several key characteristics of causal relationships: strength of association, temporality, dose response, consistency, specificity, plausibility, experiment, coherence, and analogy. Similarly, the Grading of Recommendations, Assessment, Development and Evaluation (GRADE) methodology provides a systematic approach to assessing certainty within reviews which indicates confidence that the effect estimated in evidence synthesis is close to the true effect (i.e., the causal effect).^12^ While GRADE is not always thought of as a CAA, it has been argued that it incorporates many aspects of the BH viewpoints^13^ such as incorporating risk of bias, indirectness and confounding.^14^

Other CAAs may be explicitly based on the counterfactual definition of causality. The ‘fundamental issue in causal inference’ of missing, unobserved data means that investigators cannot determine the difference between the observed effect when the individual has been exposed to the potential cause under investigation and the unobserved counterfactual outcome had the individual not been exposed, all other things being equal.^15^ Thus, application of the counterfactual definition asks investigators to consider if the unexposed group would have the same risk of the outcome as the exposed group had they also been exposed.^6^ Direct acyclic graphs (DAGs)^16^ and sufficient component cause (SCC) models (also known as causal pies) incorporate counterfactual principles in their systematic evaluation of, among other things, confounding and multifactorial causes.^17^ Epidemiologists have argued triangulating across different CAAs may help improve evaluation of putative causal relationships.^4^, ^18^ This might be particularly valuable in population health, where randomised trials are typically not possible.

The aim of this methodological review is to understand how CAAs are incorporated into population health SRs and review of systematic reviews (RoRs). We will identify SRs/RoRs that explicitly incorporate CAAs and consider how they have implemented CAAs. We will seek to elucidate any differences in the conduct of SRs/RoRs for causal assessment and consider the implications for investigators interested in using SRs/RoRs to assess causality.

## 2 METHODS

### 2.1 Review aims and scope

In this paper we use the term ‘causal SR/RoR’ to refer to SRs/RoRs which have self‐identified as assessing causality and have explicitly incorporated a CAA. Our focus on self‐identifying SRs/RoRs that have explicitly incorporated a CAA is largely due to resource and time constraints. SRs/RoRs were included if they referred to causal assessment in the title or abstract and explicitly applied CAA in the main text. Therefore, we will likely not identify SRs/RoRs that use elements of CAA but do not explicitly refer to it in the title or abstract. However, as the overall aim is to gain a broad understanding of how CAAs are incorporated into population health SRs/RoRs and offer insight into the variation for researchers wanting to conduct a causal SR/RoR, we believe the SRs/RoRs identified within this aim will provide that.

For the purposes of this review, CAA refers to the plans and procedures applied by investigators and may include any guideline, framework, tool or method used by investigators to assess causality.^19^ Some CAA examples include BH viewpoints, DAGs, GRADE or causal pies. CAAs may be informed implicitly and explicitly, and to varying degrees, by investigators' philosophical worldviews, study designs and research methodology.^19^ The assumptions about a causal relationship may be viewed through a variety of frameworks including, but not limited to: deterministic (an exposure is expected to always produce the outcome and the outcome does not occur without the exposure); probabilistic (an exposure increases the likelihood of an outcome); or multifactorial (an exposure may be a component of a complex cause that is sufficient, but not necessary, to produce the outcome).^20^, ^21^ For the purposes of this methodological review, we are agnostic under which frameworks authors were operating.

A methodological review analyses study methods.^22^ The aim of this methodological review is to identify and describe the various approaches to assessing causality in public/population health SRs/RoRs. We focus on population health, both because of its importance and the challenges in elucidating causal relationships due to the complex relationship structures and reliance on NRSs.^4^

Our aim to consider the ways in which CAAs are incorporated into population health SRs and RoRs was addressed using three objectives:

1. What CAAs have been incorporated into population health SRs?
  This objective aims to identify and describe the explicitly incorporated CAAs of self‐identifying SRs/RRs. We will note any themes of CAA characteristics that emerge.
2. How have CAAs been implemented in SRs?
  This objective aims to narratively describe how CAAs are implemented. We will highlight differences and similarities in how different CAAs are implemented and, if possible, how the same CAAs are implemented across different SRs/RoRs.
3. How were the methods for conducting causal SRs modified to incorporate the CAA?
  This objective summarises the ways SR stages are adapted to either identify evidence relevant to the CAA or analyse evidence specific to the CAA.

### 2.2 Stage 2: Identifying relevant studies

#### 2.2.1 Eligibility criteria

The eligibility criteria for this methodological review were developed according to a protocol for mixed methods: sample, phenomenon of interest, design, evaluation and research type (SPIDER).^23^ We excluded ‘research type’ due to limited relevance to our research aims. Because of the variety in CAAs and because we are not limiting our search to specific interventions or outcomes, SPIDER was deemed more appropriate than a protocol based on population, intervention, comparison and outcome (PICO).^24^ Explanations and justifications of how each protocol category and the corresponding inclusion and exclusion criteria are summarised in Table 1.

**TABLE 1:** Inclusion and exclusion criteria for reviews adapted from sample, phenomenon of interest, design, evaluation, and research type (SPIDER) protocol for mixed method studies^23^, ^92^

Protocol category	Explanation		Criteria	
	Explanation based on (34, 36)	Operationalised in methodological review	Include	Exclude
Sample	Similar to ‘population’ in PICO, sample refers to the participants included in the studies	The sample in this methodological review refers to the remit of the reviews, rather than the samples of the studies within those reviews. The remit is population or public health. There are no restrictions on criteria related to the study design, conditions, characteristics or settings within the reviews	Population health research (including public health interventions, health policy interventions, exposures such as risk factors and determinants). We focus on population health due to the importance of causal assessment in understanding both complex health interventions as well as potential health risks^4^	Clinical interventions (including pharmaceutical, surgical or psychological interventions)
Phenomenon of interest	Phenomenon of interest relates to the aim or focus of the included reviews	The phenomena of interest are reviews that explicitly stated their aim was to identify a causal relationship, including those that identified putative pathways for a causal relationship	Reviews that explicitly stated they aimed to identify a putative causal relationship between exposure and population health outcome. We focus on explicit evaluations of causality largely due to time and resource constraints	Explicit mention that likelihood of causal relationship was not considered
Design	Design refers to the study design (including any theoretical frameworks) used to inform the research methods	The review design will be limited to systematic reviews (SRs) and reviews of SRs (RoRs)	SRs and RoRs. RoRs are also included because they use similar methods and often aspire to achieve the transparency of SRs17. Because of the variation in how SRs/RoRs are defined, we defined inclusion by self‐identification. Determining how closely a review followed SR/RoR principles and methods was beyond the scope of this methodological review	Non‐systematic reviews including methodological review
Evaluation	The term ‘evaluation’ is comparable to ‘outcomes’ in PICO. To accommodate qualitative research, evaluation includes unmeasurable findings	For the purposes of this methodological review, this refers to what approaches are incorporated into the reviews	Reviews that have incorporated approach(es) to causal assessment. We limited SRs/RoRs to those that explicitly incorporated one or more causal assessment approach (CAA) because of resources and time constraints	No explicit mention approach has been incorporated to support causal assessment
Research type	The research type refers to either quantitative, qualitative or mixed methods	This protocol category was not utilised as we did not have restrictions related to criteria for study design of the review	Not applicable	Not applicable

Abbreviation: PICO, population, intervention, comparison and outcome.

##### Full list of exclusion criteria

1. Reviews of clinical intervention or evaluation studies or other studies not related to population or public health.
2. Reviews that do not self‐identify as having conducted or considered causal assessment.
3. Reviews that do not self‐identify as a SR or RoR.

We excluded reviews that hypothesised, but did not evaluate, possible causal mechanisms, links or pathways,^25^, ^26^, ^27^, ^28^, ^29^ or reviews that included studies that aimed to assess, or stated that they had assessed, causality but did not implement any causal assessment (see Table 1).

#### 2.2.2 Search strategy

The goal of the search was to identify SRs and RoRs in population health that assess causality. We identified reviews in a systematic search of three electronic bibliographic databases conducted in February 2020: EMBASE, Medline, and CINAHL. Our search included keywords related to ‘systematic review’ and ‘causality’ in the title and abstract and, where possible, as subject headings. To limit the search to SRs, one of our key terms was the subject heading, ‘systematic review’. We also included terms such as ‘causal’ or ‘causation’ or ‘causal assessment’ or ‘causal evaluation’ in the title or abstract. As we focused on recent practice in SRs and RoRs used for causal assessment, our search was limited to January 2000–February 4, 2020. The reviews were further limited to English language reviews and the population in our search were limited to human subjects. The research team finalised the search strategy in consultation with an information specialist (see Appendix A for full search strategy).

### 2.3 Stage 3: Study selection

Following de‐duplication using Covidence, titles and abstracts were exported to EndNote X9 © and screened in two stages: (1) title and abstract and (2) full‐text. At both stages, reviews were independently reviewed by two investigators (MS and: HT, SVK, or AP). A third reviewer was consulted about disagreements at either stage.

#### 2.3.1 Data extraction

Data extraction was completed by MS. A second reviewer (HT, SVK or AP) checked a 10% sample of purposively selected reviews that spanned a range of different CAA and provided good coverage all the potential issues that might arise. As most of the outcomes were qualitative descriptions of methods rather than statistical estimates, we did not calculate specific interrater reliability measures. Rather, we aimed to explore interpretation of phenomena through discussion as is common in qualitative research, particularly focusing on non‐BH CAA methods.^30^ The data extraction form (see Appendix B) included both structured and free‐text domains and was piloted before finalising. We extracted data on key study information such as type of review, study designs included in the review, and PICO features as well as which CAA was used (e.g., BH viewpoints), key features of causal approaches (e.g., identifying confounders, temporality, etc.) and criteria used to meet each CAA (e.g., specific study design).

#### 2.3.2 Data summary and synthesis

The data were tabulated to facilitate comparison across SRs/RoRs that used a particular CAA as well as comparison across reviews that incorporated different CAAs. The data for each CAA were then summarised narratively to describe the variations in how CAAs were implemented. We tabulated the following information which was considered to be quantifiable: the number of reviews that used each CAA; which BH viewpoints were used; if the viewpoints were defined; how authors determined if viewpoints were met (or in other words, did they identify and apply indicators); how overall support for viewpoints was determined; and how the viewpoints were applied (Table 3). We thematically collated free‐text responses, such as the impact of causal approach on SR/RoR stages (Table 4), where possible. Both this ‘quantifiable’ information and other qualitative information were synthesised descriptively.

## 3 RESULTS

### 3.1 Included reviews

**FIGURE 1:**
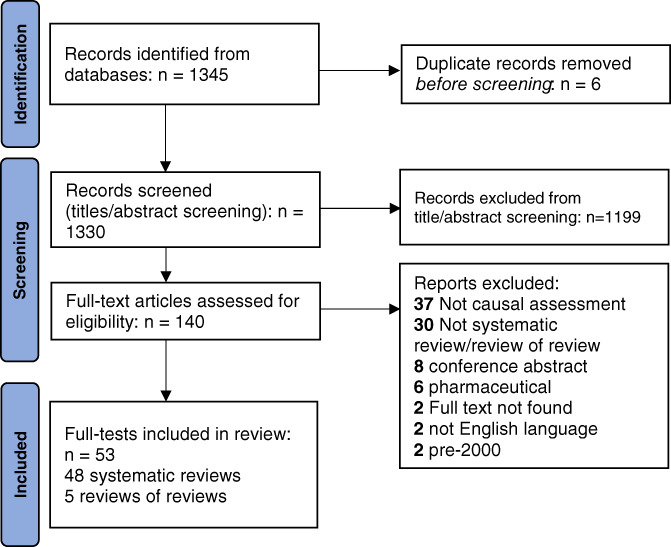
PRISMA flow diagram with primary reasons for excluding full text reviews [Colour figure can be viewed at wileyonlinelibrary.com]

Figure 1 shows the flowchart of the searches.^31^ The search resulted in 1345 references. Out of 1339 de‐duplicated screened references, 140 full texts were assessed and 53 reviews were included (five were RoRs,^32^, ^33^, ^34^, ^35^, ^36^ all of which used BH viewpoints).

### 3.2 CAAs

The review characteristics, including the exposure topic area, CAA(s) used, and critical appraisal tool(s) applied by the review are provided in Appendix C. Forty‐six reviews (46/53, 86.7%) applied BH viewpoints,^32^, ^33^, ^34^, ^35^, ^36^, ^37^, ^38^, ^39^, ^40^, ^41^, ^42^, ^43^, ^44^, ^45^, ^46^, ^47^, ^48^, ^49^, ^50^, ^51^, ^52^, ^53^, ^54^, ^55^, ^56^, ^57^, ^58^, ^59^, ^60^, ^61^, ^62^, ^63^, ^64^, ^65^, ^66^, ^67^, ^68^, ^69^, ^70^, ^71^, ^72^, ^73^, ^74^, ^75^, ^76^, ^77^ with a further seven using ‘alternative’ approaches: two (2/53, 3.8%) incorporated the Medical Research Council (MRC) guidance on natural experiments^78^, ^79^; two reviews (2/53, 3.8%) utilised realist reviews as a CAA^80^, ^81^; one (1/53, 1.9%) utilised horizontal SRs^82^; one (1/53, 1.9%) incorporated ‘sign testing’ of causal mechanisms^83^; and another one (1/53, 1.9%) used a causal cascade model.^84^

The complete list of CAAs identified (objective 1) and descriptions of how CAAs were implemented (objective 2) can be found in Table 2. We provide additional detail comparing implementation of BH viewpoints in Table 3. Because most other CAAs were only used by one or two reviews, we were only able to compare implementation for BH viewpoints. A comparison of how realist reviews and MRC guidance on natural experiments were implemented was described narratively in Sections 3.2.2 and 3.2.3.

**TABLE 2:** Overview of description of causal assessment approaches (CAAs) and how they were incorporated into systematic reviews (SRs) and reviews of reviews (RoRs)

CAA	Number of reviews	Description of CAA	How CAA was incorporated into SRs
Bradford Hill (BH) viewpoints	46^32^, ^33^, ^34^, ^35^, ^36^, ^37^, ^38^, ^39^, ^40^, ^41^, ^42^, ^43^, ^44^, ^45^, ^46^, ^47^, ^48^, ^49^, ^50^, ^51^, ^52^, ^53^, ^54^, ^55^, ^56^, ^57^, ^58^, ^59^, ^60^, ^61^, ^62^, ^63^, ^64^, ^65^, ^66^, ^67^, ^68^, ^69^, ^70^, ^71^, ^72^, ^73^, ^74^, ^75^, ^76^, ^77^	BH viewpoints, also known as criteria, are a set of nine characteristics to consider when assessing a causal relationship.^2^ The nine viewpoints are: strength of association, consistency, specificity, temporality, dose–response, plausibility, coherence, experiment, and analogy	The most commonly used CAA, there was considerable variation in which BH viewpoints were used and how they were operationalised. There was also variation in transparency and clarity about how the viewpoints were incorporated and used in causal assessment
Medical research council (MRC) guidance on natural experiments	2^78^, ^79^	The MRC guidance on natural experiments posits that certain study designs and analytic methods are more suitable to assess causality than others, and suggests that results from different studies be compared.^85^ The MRC guidance on natural experiments highlights study design, including carefully defining control groups to establish exchangeability with exposed individuals and testing underpinning methodological assumptions as important for establishing causality. It also draws attention to some methods (such as difference‐in‐differences, regression discontinuity designs, and instrumental variable analysis) which can address unmeasured, as well as measured, confounders	Two reviews^78^, ^79^ used MRC guidance on natural experiments.^85^ One limited their scope to studies that incorporated methods deemed to be of high quality in MRC guidance on natural experiments, while the other review considered study design and analytic methods during evidence synthesis
Realist reviews	2^80^, ^81^	Realist reviews are an established CAA with an existing set of guidelines for incorporating realist synthesis principles into SRs.^86^ One of the main aims of realist reviews is understand contexts and reasons for a causal relationship. Realist reviews often incorporate different forms of evidence, including theoretical evidence	Both realist reviews narratively assessed causal mechanisms that may explain the relationship under study, and both determined that further evidence is needed to understand possible mechanisms
Horizontal systematic review	1^82^	This CAA was developed by the review authors to collate evidence of causal effects across a range of study designs and risk (identified for having varying properties, such as threat of confounding, measurement error or proximity to the outcome on the causal pathway	The authors considered evidence from observational studies (that accounted for confounding and reverse causation), genetic studies using Mendelian randomisation, and RCTs for four risk factors Separate meta‐analyses were conducted for each risk factor and by study design. The meta‐analysis results were compared across risk factors, considering the differing sources and level of bias across the different methods
Sign test hypotheses	1^83^	This approach, interrogates the evidence for reverse causation (such that the outcome is in fact the cause of the exposure)	The authors^83^ interrogated the evidence for two the direction of causation between the exposure and outcome to establish whether there was evidence for one direction being stronger than the other
Causal cascade method	1^84^	Based on logic model developed to illustrate the ‘framework of causal relationships’, the authors conducted a Bayesian meta‐analyses on the heterogeneity across RCTs	Authors hypothesised reasons for heterogeneity found in RCTs evaluating breast cancer screening on mortality—including attendance rates, the accuracy of screening tests, and social class. The logic model in Figure 1 of the review illustrates the framework of causal relationship and includes the key cascade components (attendance rates and sensitivity) that may account for differences in two outcomes (advanced breast cancer and breast cancer mortality). The authors then considered the trial evidence across these different inter‐related factors to consider whether heterogeneity in the evidence base could be explained by these factors. Based on the assumptions in the logic model and the included studies, the review estimated the relative risk of advanced‐stage breast cancer and breast cancer mortality by three different attendance rates and sensitivity in trials (a total of nine scenarios). Overall, they found that attendance rate and sensitivity may explain statistical heterogeneity across trials

*Note*: The review topics, in terms of exposures, varied: sixteen (16/53, 30.2%) reviews focused on occupational health^32^, ^33^, ^34^, ^35^, ^60^, ^61^, ^62^, ^63^, ^64^, ^65^, ^69^, ^70^, ^73^, ^74^, ^75^; eleven (11/53, 20.8%) on environmental health^40^, ^44^, ^46^, ^47^, ^49^, ^59^, ^68^, ^71^, ^78^, ^79^; nine (9/53, 17.0%) on nutritional health^43^, ^51^, ^52^, ^53^, ^54^, ^55^, ^57^, ^77^, ^81^; four (4/53, 7.5%) on smoking^42^, ^45^, ^66^, ^72^; four (4/53, 7.5%) on mental health^48^, ^56^, ^67^, ^82^; three (3/53, 5.7%) on alcohol consumption^36^, ^38^, ^39^; two (2/53, 3.8%) on child health^50^, ^58^; two (2/53, 3.8%) on health inequalities^41^, ^83^; one (1/53, 1.9%) on diagnostics^84^; and one (1/53, 1.9%) on respiratory diseases.^37^

Abbreviation: RCTs, randomised controlled trials.

**TABLE 3:** Overview of how Bradford Hill (BH) viewpoints were applied, categorised by five domains

Domain	Description	Summary of results
Viewpoints used	The viewpoints used in each review	Strength of association: 44/46, 95.7%^32^, ^33^, ^34^, ^35^, ^36^, ^37^, ^38^, ^39^, ^40^, ^41^, ^42^, ^43^, ^44^, ^45^, ^46^, ^47^, ^48^, ^49^, ^50^, ^51^, ^52^, ^53^, ^54^, ^56^, ^57^, ^58^, ^59^, ^60^, ^61^, ^62^, ^63^, ^64^, ^65^, ^66^, ^67^, ^69^, ^70^, ^71^, ^72^, ^73^, ^74^, ^75^, ^76^, ^77^ Temporality: 44/46, 95.7%^32^, ^33^, ^34^, ^35^, ^36^, ^37^, ^38^, ^39^, ^40^, ^41^, ^42^, ^43^, ^44^, ^46^, ^48^, ^49^, ^50^, ^51^, ^52^, ^53^, ^54^, ^55^, ^56^, ^57^, ^58^, ^59^, ^60^, ^61^, ^62^, ^63^, ^64^, ^65^, ^66^, ^67^, ^68^, ^69^, ^70^, ^71^, ^72^, ^73^, ^74^, ^75^, ^76^, ^77^ Dose–response: 43/46, 93.5%^32^, ^33^, ^34^, ^35^, ^36^, ^37^, ^38^, ^39^, ^40^, ^41^, ^42^, ^43^, ^44^, ^46^, ^47^, ^48^, ^49^, ^50^, ^51^, ^52^, ^53^, ^55^, ^56^, ^57^, ^58^, ^59^, ^60^, ^61^, ^62^, ^63^, ^64^, ^65^, ^66^, ^68^, ^69^, ^70^, ^71^, ^72^, ^73^, ^74^, ^75^, ^76^, ^77^ Consistency: 41/46, 89.1%^32^, ^33^, ^34^, ^35^, ^37^, ^38^, ^39^, ^40^, ^41^, ^42^, ^43^, ^44^, ^46^, ^47^, ^49^, ^50^, ^51^, ^52^, ^53^, ^54^, ^56^, ^57^, ^58^, ^59^, ^60^, ^61^, ^62^, ^63^, ^64^, ^65^, ^66^, ^68^, ^69^, ^70^, ^71^, ^72^, ^73^, ^74^, ^75^, ^76^, ^77^ Plausibility: 38/46, 82.6%^33^, ^34^, ^36^, ^37^, ^38^, ^39^, ^40^, ^41^, ^42^, ^43^, ^44^, ^46^, ^47^, ^49^, ^52^, ^53^, ^55^, ^56^, ^57^, ^58^, ^59^, ^60^, ^61^, ^62^, ^63^, ^64^, ^65^, ^66^, ^68^, ^69^, ^70^, ^71^, ^72^, ^73^, ^74^, ^75^, ^76^, ^77^ Experiment: 32/46, 69.6%^33^, ^34^, ^35^, ^36^, ^38^, ^39^, ^40^, ^41^, ^43^, ^45^, ^46^, ^52^, ^53^, ^55^, ^57^, ^59^, ^60^, ^61^, ^62^, ^63^, ^64^, ^65^, ^67^, ^68^, ^69^, ^70^, ^71^, ^72^, ^73^, ^74^, ^75^, ^76^ Coherence: 21/46, 45.7%^33^, ^34^, ^38^, ^39^, ^40^, ^41^, ^42^, ^46^, ^47^, ^52^, ^53^, ^54^, ^55^, ^57^, ^59^, ^65^, ^69^, ^70^, ^71^, ^72^, ^76^ Specificity: 18/46, 39.1%^33^, ^34^, ^38^, ^39^, ^40^, ^41^, ^46^, ^52^, ^53^, ^55^, ^59^, ^65^, ^68^, ^69^, ^70^, ^71^, ^72^, ^76^ Analogy: 15/46, 32.6%^38^, ^40^, ^41^, ^42^, ^46^, ^52^, ^53^, ^55^, ^59^, ^65^, ^69^, ^70^, ^71^, ^72^, ^76^
Viewpoint definition	Whether a description, interpretation or definition of each viewpoint is provided	Description,/interpretation/definition provided: 15/46, 32.6%^32^, ^33^, ^34^, ^35^, ^38^, ^43^, ^46^, ^53^, ^54^, ^55^, ^57^, ^58^, ^70^, ^72^, ^76^
Viewpoint indicators	Criteria to determine if viewpoint had been met are reported	Indicators used and reported: 19/46, 41.3%^32^, ^38^, ^42^, ^45^, ^46^, ^50^, ^52^, ^53^, ^54^, ^55^, ^60^, ^61^, ^62^, ^63^, ^64^, ^70^, ^73^, ^74^, ^75^ Example of indicators include, but are not limited to, quantitative ranges (e.g., risk ratio (RR) or odds ratio (OR) between 3.0 and 8.0 for strong association) or qualitative thresholds (e.g., at least one credible mechanism to explain association for plausibility)
Overall support for viewpoints	Report level or degree of support for each viewpoint (e.g., strong, moderate, and weak)	44/46 (95.7%)^32^, ^33^, ^34^, ^35^, ^36^, ^37^, ^38^, ^39^, ^40^, ^41^, ^42^, ^43^, ^44^, ^45^, ^46^, ^47^, ^49^, ^50^, ^51^, ^52^, ^53^, ^54^, ^55^, ^56^, ^57^, ^58^, ^59^, ^60^, ^61^, ^62^, ^63^, ^64^, ^66^, ^67^, ^68^, ^69^, ^70^, ^71^, ^72^, ^73^, ^74^, ^75^, ^76^, ^77^ reviews described support for each viewpoint to be met. This was done narratively, quantitatively (e.g., number of studies in support of viewpoint; probability that viewpoint was met) or some combination of both
Viewpoint application	Viewpoints were applied before or after evidence was synthesised and could be applied to all studies as a collective, studies individually or groups of studies (e.g., by study design, by exposure/outcome relationship). Some reviews applied viewpoints in more than one way	36/46 (78.3%)^33^, ^34^, ^36^, ^38^, ^39^, ^40^, ^42^, ^43^, ^44^, ^45^, ^46^, ^49^, ^50^, ^51^, ^52^, ^53^, ^54^, ^55^, ^56^, ^57^, ^58^, ^59^, ^60^, ^61^, ^62^, ^66^, ^67^, ^68^, ^69^, ^70^, ^71^, ^72^, ^73^, ^74^, ^75^, ^76^ reviews assessed each BH viewpoint by applying them across the evidence (i.e., after studies were synthesised with studies considered collectively) 13/46 (28.3%)^35^, ^37^, ^40^, ^41^, ^43^, ^48^, ^53^, ^54^, ^55^, ^58^, ^60^, ^62^, ^64^ applied the viewpoints across the exposure/outcome relationship(s) under study (i.e., after studies were synthesised, but with studies or different exposure/outcomes considered individually) 12/46 (26.1%)^35^, ^38^, ^48^, ^54^, ^60^, ^61^, ^62^, ^63^, ^64^, ^66^, ^73^, ^74^, ^75^ applied the viewpoints to each included study (i.e., before synthesis with studies considered separately) 4/46 (8.7%)^32^, ^54^, ^57^, ^58^ reviews assessed if viewpoints were met by applying them separately to different study designs 1/46 (2.2%) review^47^ applied the viewpoints to studies deemed high‐quality only It was unclear how 2/46 (4.3%) reviews^65^, ^77^ applied viewpoints to evidence

*Note*: The domain descriptions and corresponding reviews are summarised.

#### 3.2.1 BH viewpoints

While the majority of reviews applied BH viewpoints to assess causality, there was considerable variation in how they were implemented. As described in Section 2.3.1, we extracted information to evaluate how implemention of BH viewpoints varied which we categorise into five key domains: (1) viewpoints used; (2) viewpoint definition; (3) viewpoint indicators (i.e., how was the viewpoint assessed as being ‘met’); (4) assessment of overall support for viewpoints; and (5) if viewpoints were considered across the body of evidence or in another way (e.g., across a single study or relationship). An overview of each domain can be found in Table 3.

##### BH viewpoints assessed

Twelve (12/46, 26.1%)^38^, ^40^, ^41^, ^46^, ^52^, ^53^, ^55^, ^59^, ^65^, ^69^, ^70^, ^71^, ^72^, ^76^ SRs/used all nine BH viewpoints. Coherence, specificity and analogy were the least commonly assessed, featuring in fewer than half of the reviews. Three (3/46, 6.5%) SRs^33^, ^34^, ^57^ combined coherence and plausibility. In all but three SRs (3/46, 4.3%)^41^, ^53^, ^57^ it was unclear why certain viewpoints were excluded.

##### Definitions of BH viewpoints

Clearly defining viewpoints is important for transparent implementation of BH viewpoints, but fewer than half (16/46, 34.8%) of reviews^32^, ^33^, ^34^, ^35^, ^36^, ^38^, ^43^, ^46^, ^53^, ^54^, ^55^, ^57^, ^58^, ^70^, ^72^, ^76^ did so explicitly and the definitions varied. For example, consistency was defined by Fenton and colleagues^43^ as variation across different study designs while others^32^, ^33^, ^34^, ^35^, ^57^ described consistency as variation across populations and settings. Norman and colleagues defined consistency as observing a comparable association across various study designs, populations, settings and regions.^58^ Livesey and colleagues were the only review to consider sources of heterogeneity when evaluating consistency across studies.^53^

##### Indicators used for meeting BH viewpoints

Viewpoint indicators (i.e., criteria to determine if viewpoints are met) are useful for understanding differences in how BH viewpoints were used in causal assessment. However, fewer than half (19/46, 41.3%) of reviews reported what criteria were used to determine if a viewpoint was met. For those that did provide indicators, there was considerable variation. For example, the indicators for assessing strength of association, though all quantitative, varied widely: risk ratio (RR) greater than 1.20^52^; RR greater than 0.9 for protective factors and greater than 1.25 for harmful factors^55^; RR or odds ratio (OR) between 3.0 and 8.0^42^; hazard ratio (HR) greater than or equal to 3.0^45^; OR greater than 4.0^50^, ^60^, ^61^, ^62^, ^63^, ^64^, ^73^, ^74^, ^75^; RR greater than 5.0^46^; or a greater than 10% increased risk and statistically significant.^32^ None of the reviews that provided indicators for strength of association considered confounding adjustment, including residual or unmeasured confounding, when assessing whether strength of association was met. This is important as bias may fully explain a large association (and small associations may not entirely explained by bias). However, some of the reviews (9/46, 19.6%)^44^, ^47^, ^49^, ^50^, ^56^, ^58^, ^59^, ^66^, ^69^ broadly considered the findings from individual studies or their findings when evidence was synthesised in the context of confounding and bias, which in some reviews was also referred to as ‘alternative explanations’.

While strength of association relied on quantitative indicators, some indicators for other viewpoints were less definitive. Five reviews^38^, ^42^, ^46^, ^52^, ^55^ provided indicators for the plausibility viewpoint (out of 38 reviews that included plausibility in their assessment). Two reviews^52^, ^55^ determined that plausibility was met if at least one credible, hypothetical mechanism explained the association (e.g., empirical studies demonstrating a relationship), though neither clarified what was meant by ‘credible’. Similarly, Hughes and colleagues determined that the relationship under study was plausible if there were positive animal or mechanistic data.^46^ On the other hand, rather than focus on hypothetical explanations for an association, two other reviews^38^, ^42^ noted that an association between the exposure and outcome under study in human studies was sufficient evidence for plausibility. None of the SRs/RoRs explained why certain indicators were used, making it challenging to discern the underlying reasons for the variation of indicators used for a given viewpoint (e.g., range of indicators for strength of association).

##### Support for BH viewpoints

Viewpoint indicators describe the necessary criteria to determine *if* each viewpoint was met (at the study level), while the overall support for each viewpoint reflects the *extent* to which each viewpoint was met (based on the body of evidence). Most reviews (44/46, 95.7%)^32^, ^33^, ^34^, ^35^, ^36^, ^37^, ^38^, ^39^, ^40^, ^41^, ^42^, ^43^, ^44^, ^45^, ^46^, ^47^, ^49^, ^50^, ^51^, ^52^, ^53^, ^54^, ^55^, ^56^, ^57^, ^58^, ^59^, ^60^, ^61^, ^62^, ^63^, ^64^, ^66^, ^67^, ^68^, ^69^, ^70^, ^71^, ^72^, ^73^, ^74^, ^75^, ^76^, ^77^ set out to assess the level of support provided in the evidence reviewed for the viewpoints (or viewpoint indicator, where applicable) being met. Two (2/46, 4.3%) reviews^48^, ^65^ did not report whether the level of support for the viewpoints was or was not met. Assessments were done narratively or quantitatively, with seventeen reviews (17/44, 38.6%)^32^, ^33^, ^34^, ^38^, ^41^, ^43^, ^45^, ^46^, ^52^, ^54^, ^60^, ^61^, ^66^, ^70^, ^73^, ^74^, ^75^ using both narrative and quantitative approaches to assess the support for viewpoints being met. Seventeen (17/44, 38.6%) reviews^36^, ^37^, ^39^, ^44^, ^47^, ^49^, ^50^, ^51^, ^53^, ^56^, ^57^, ^59^, ^67^, ^68^, ^69^, ^71^, ^76^ provided narrative‐only assessments. Most SRs/RoRs (27/44, 61.4% of those that assessed support for viewpoints) included at least one quantitative assessment including:

- Ordinal assessment
  - strong/moderate/weak: fourteen reviews (14/44, 31.8%)^33^, ^34^, ^38^, ^42^, ^45^, ^58^, ^60^, ^61^, ^62^, ^63^, ^64^, ^73^, ^74^, ^75^
  - yes, no, strong, poor, none (1/44, 2.3%)^70^
  - conclusive, inconclusive, null (1/44, 2.3%)^32^
  - +++ evidence from several well‐designed studies, ++ evidence from several studies but with important limitations; + emerging evidence from a few studies or conflicting results from several studies, — criterion not met (1/44, 2.3%)^55^
  - supportive, not applicable/not examined, no association/negative (1/44, 2.3%)^66^
  - high, moderate, doubtful/low, unclear (1/44, 2.3%)^72^
- Binary assessment
  - yes/no (5/44, 11.4%)^40^, ^43^, ^52^, ^54^, ^77^
- Other
  - number of studies that supported each viewpoint when assessing if the viewpoint was met two reviews (2/44, 4.5%)^35^, ^41^
  - probability that each viewpoint was met (1/44, 2.3%)^46^

##### Application of BH viewpoints to evidence

Most SRs/RoRs using BH viewpoints (36/46, 78.3%),^33^, ^34^, ^36^, ^38^, ^39^, ^40^, ^42^, ^43^, ^44^, ^45^, ^46^, ^49^, ^50^, ^51^, ^52^, ^53^, ^54^, ^55^, ^56^, ^57^, ^58^, ^59^, ^60^, ^61^, ^62^, ^66^, ^67^, ^68^, ^69^, ^70^, ^71^, ^72^, ^73^, ^74^, ^75^, ^76^ assessed each BH viewpoint by applying them across the body of evidence (i.e., after studies were synthesised with studies considered collectively) while another thirteen (13/46, 28.2%) reviews^35^, ^37^, ^40^, ^41^, ^43^, ^48^, ^53^, ^54^, ^55^, ^58^, ^60^, ^62^, ^64^ applied the viewpoints across the exposure/outcome relationship(s) under study (i.e., after studies were synthesised, studies considered collectively by exposure/outcome relationship). Twelve SRs/RoRs^35^, ^38^, ^48^, ^54^, ^60^, ^61^, ^62^, ^63^, ^64^, ^66^, ^73^, ^74^, ^75^ applied the viewpoints to each included study (i.e., before synthesis with studies considered separately). Four (4/46, 8.7%) SRs/RoRs^32^, ^54^, ^57^, ^58^ applied viewpoints separately to different study designs, while one (1/46, 2.2%) SR^47^ applied the viewpoints only to studies deemed higher quality in terms of causal assumptions. It was unclear how two reviews^65^, ^77^ applied viewpoints on evidence.

#### 3.2.2 MRC guidance on natural experiments

Two SRs^78^, ^79^ used the MRC guidance on natural experiments^85^ to conduct causal assessment. This CAA involves identifying observational studies that appropriately and comprehensively address bias, deeming them most suitable to assess causality. The guidance focusses predominantly on natural experiment study designs and other analytical approaches that compare outcomes pre‐ and post‐intervention, partly to discern if the exposure preceded the outcome. The guidance favours analytical methods that address observable and measurable sources of bias from confounding (e.g., matching, regression adjustment, and propensity scores) and unmeasured or residual confounding (e.g., differences in differences, instrumental variables, and regression discontinuity).

Martin and colleagues^78^ identified studies that consider the relationship between the built characteristics of an environment and obesity that applied any of the analytical methods described by the MRC guidance on natural experiments to address observable or unobservable confounders.^78^ They found that the observed associations in studies using methods to address particular sources of bias (e.g., longitudinal studies which are more suitable to consider the temporal ordering of variables) were comparable with those that did not (such as cross‐sectional studies, which cannot always establish temporality). The comparable results appear to increase the validity of observational studies in determining strength of association.

Molenberg and colleagues,^79^ on the other hand, did not limit their search to studies that incorporated these analytical methods. Instead, they extracted evidence from the included studies that, based on the MRC guidance on natural experiments, used methods that may help elucidate the possible causal relationship between infrastructural intervention to promote cycling and cycling outcomes. Specifically, they noted which studies considered multiple comparison groups to test robustness of findings (e.g., infrastructural intervention on cycling for cyclists vs. non‐cyclists) and the use of complementary research methodologies (e.g., trends from surveys). They aimed to also consider the effect of changes in the infrastructural intervention on a neutral outcome that is expected to be independent from the intervention (i.e., a falsification outcome), though did not identify any studies that used falsification outcomes. Thus, this application of the MRC guidance on natural experiments appears to reflect the principles of three BH viewpoints: temporality (focusing on study designs that ensure the exposure preceded the outcome), experiment (study designs focus on comparing pre‐ and post‐intervention), and specificity (falsification outcomes).

#### 3.2.3 Realist reviews

A realist review is an evidence synthesis strategy used to investigate the context and mechanisms through which an exposure‐outcome relationship operates.^86^ Realist reviews aim to provide a more iterative approach to examining complex interventions than traditional SRs, which have been criticised for being too inflexible. In doing so, the included realist reviews appear to focus on the BH viewpoint of plausibility. Two SRs utilised the realist review approach to assess causality.^80^, ^81^ DeBono and colleagues evaluated the relationship between participation in the US food stamp programme and obesity^81^ while Blair and colleagues^80^ applied a realist review to understand the causal mechanism through which neighbourhood impact depression. Both SRs underscored the goal of realist reviews to explore and explain the causal mechanism of the relationship under study, which both SRs did in part by extracting the posited causal pathways from the included studies and then narratively assessing the evidence for different pathways. Neither SR found strong evidence for any of the proposed mechanisms.

#### 3.2.4 Horizontal SR

Kuper and colleagues implemented a novel approach to causal assessment across a body of evidence for a range of risk factors which they called a ‘horizontal SR’. They examined the relationship between four risk factors (depression, exercise, C reactive protein, and diabetes) and coronary heart disease, using various study designs.^82^ Within and across the risk factors, they compared findings across study designs which addressed confounding and reverse causality to different degrees and in different ways: observational studies with multivariable adjustments; studies using genetic variants as instrumental variables (Mendelian randomisation); and RCTs.^82^

For each risk factor, they conducted a meta‐analysis by each study design type and subsequently compared the meta‐analysis results of the three risk factors with an unknown causal role (depression, exercise, and C reactive protein) against the meta‐analysis results of the risk factor they designated an established cause (diabetes). The comparison of observational studies suggested that diabetes and C reactive protein had a causal role in coronary heart disease, while, according to the authors, observational evidence for exercise and diabetes was more susceptible to bias and thus their causal effect on coronary heart disease was inconclusive. There was only evidence from Mendelian randomisation studies and RCTs for C reactive protein, where it appears that C reactive protein did not have a causal role, making it difficult to compare results and thus make any causal inferences.

In identifying studies that address bias and study designs comparable to experimental evidence, this CAA utilised the principles of the BH viewpoints strength of association across different study designs and experiment. Unlike the reviews applying BH viewpoints, in a horizontal review the size of association identified from the observational studies is considered in the context of appropriately account for confounding and other forms of bias. Kuper and colleagues also considered other forms of bias including measurement bias and publication bias. The authors also appear to consider specificity as by looking at different risk factors they may implicitly, and unintentionally, suggesting there is no evidence for specificity. They also appear to account for consistency within the horizontal SR as they are not only evaluating effect estimates across different study designs and risk factors, but also explicitly review explanations for statistical heterogeneity and evaluate temporality while considering reverse causality.

#### 3.2.5 Sign test hypotheses

A SR by Kroger and colleagues explored the relationship between socioeconomic status and health by comparing two competing hypotheses that could explain the putative causal relationship.^83^ The health selection hypothesis suggests that differences in health status cause socioeconomic status while the social causation hypothesis suggests that resources available to people with higher socioeconomic status have better health (i.e., reverse causality). To determine which mechanism is more likely to be causal, Kroger and colleagues conducted a sign‐test to compare the probabilities of health selection versus social causation based on the conclusions of included studies. This CAA reflects an approach to testing for temporality by testing the reverse direction of the pathways between the exposure and outcome.

The authors ran three meta‐regressions: one for all studies providing support to the health selection hypothesis; one for all studies in support of the social causation hypothesis; and one for all studies that found equal support for both hypotheses (i.e., the null hypothesis). They regressed the preference for the three theories against study characteristics including age, education and income of the included studies' samples, which were found to be somewhat predictive of support for a given theory. Overall, they did not find a consensus in support for either theory. Thus, it appears that strength of association is implemented in the context of understanding temporality. This CAA uses temporality, also used in BH viewpoints, to assess reverse causation.

#### 3.2.6 Causal cascade model

One SR implemented the principles of DAGs (‘conditional independence for the parameters and variables implicated’ p5^84^) and developed a Bayesian causal model illustrating the ‘framework of causal relationships’, p3.^84^ The model illustrated the framework of causal relationships Chen and colleagues aimed to understand the heterogeneity of advanced breast cancer risk and mortality breast cancer across breast cancer screening trials. They focused on two hypothesised reasons for variation in trials examining breast cancer mortality: attendance rate in screening trials and test sensitivity to breast cancer mammography (i.e., incidence rate of interval cancer/expected incidence rate). In other words, their aim was to elucidate the statistical heterogeneity of advanced breast cancer risk within breast cancer screening trials given these two possible explanations. They considered the impact of different combinations of attendance rates (90%, 60%, and 30%) and sensitivity rates (95%, 75%, and 55%) on breast cancer risk and mortality rates. They found that both attendance rates and sensitivity explained the heterogeneity of trials. This CAA overlaps with the BH viewpoint of consistency, which is concerned with heterogeneity across the evidence.

### 3.3 CAAs impact on conduct of SR stages

In this section we considered whether and how CAAs impacted different stages of SR conduct (objective 3): objective of the review; description of the study design; inclusion and exclusion criteria; search strategy; data extraction; and evidence synthesis and conclusion.^87^ The key findings and adaptations made in each stage are summarised below in Table 4.

**TABLE 4:** Impact of causal assessment approaches (CAAs) o conduct of systematic review (SR) stages

Review stage	Number of reviews (*n* = 53)	CAA incorporated into SR stage
Review aims and objective	39/53, 73.6%^32^, ^34^, ^35^, ^36^, ^37^, ^38^, ^42^, ^43^, ^45^, ^50^, ^51^, ^52^, ^53^, ^54^, ^55^, ^56^, ^57^, ^59^, ^60^, ^61^, ^62^, ^63^, ^64^, ^66^, ^67^, ^68^, ^69^, ^70^, ^72^, ^73^, ^74^, ^75^, ^78^, ^79^, ^80^, ^81^, ^82^, ^83^, ^84^	Most reviews explicitly stated that one of their review aims and objectives was to assess (statistically and/or narratively) evidence for causal relationship. The nature of how causality was assessed varied across reviews, where reviews might focus on statistically analysing the evidence and/or on narratively assessing the evidence
Review design	41/53, 77.4%^32^, ^34^, ^36^, ^37^, ^38^, ^40^, ^41^, ^42^, ^43^, ^45^, ^46^, ^48^, ^50^, ^51^, ^52^, ^53^, ^54^, ^55^, ^57^, ^59^, ^60^, ^61^, ^62^, ^63^, ^64^, ^65^, ^66^, ^68^, ^69^, ^70^, ^72^, ^73^, ^74^, ^75^, ^76^, ^78^, ^80^, ^81^, ^82^, ^83^, ^84^	Most reviews included their CAA as a specific part of their overall review design for identifying, synthesising, and analysing evidence. Including CAA in a SR study design suggests that causal assessment was an a priori consideration for the SR, an important fact to consider when critically appraising SRs^93^ (which was beyond the scope of this review)
Inclusion/exclusion criteria	14/53, 26.4%^45^, ^60^, ^61^, ^62^, ^64^, ^68^, ^73^, ^74^, ^75^, ^78^, ^80^, ^82^, ^83^, ^84^	Of the 14 reviews that designed criteria to reflect incorporating CAAs, 10 reviews (all of which utilised BH viewpoints except for one realist review) included studies that considered potential causal pathways^60^, ^61^, ^62^, ^64^, ^68^, ^73^, ^74^, ^75^, ^80^ or excluded studies that did not.^45^ Another four reviews^43^, ^78^, ^82^, ^83^ (all but one using alternative CAAs) limited studies to those deemed most useful for assessing causality: observational studies utilising analytical methods to account for confounding, study designs that provide experimental evidence; and studies testing two competing causal mechanisms
Search terms	3/53, 5.7%^43^, ^74^, ^78^	A few reviews designed their search strategies to specifically identify studies that support their CAA. Common terms included: causality, experiment, instrumental variable, regression discontinuity, and mechanism
Data extraction	14/53, 26.4%^32^, ^35^, ^42^, ^44^, ^50^, ^60^, ^61^, ^69^, ^74^, ^79^, ^80^, ^81^, ^83^, ^84^	All 14 reviews^32^, ^35^, ^42^, ^44^, ^50^, ^60^, ^61^, ^74^ extracted information that supported the CAA such as the confounding variables that were conditioned upon in their included studies, study design information that may strengthen causal inference or possible causal mechanisms
Synthesis	51/53, 96.2%^32^, ^33^, ^34^, ^35^, ^36^, ^37^, ^38^, ^39^, ^40^, ^41^, ^42^, ^43^, ^44^, ^45^, ^46^, ^47^, ^48^, ^49^, ^50^, ^51^, ^52^, ^53^, ^54^, ^55^, ^56^, ^57^, ^58^, ^59^, ^60^, ^61^, ^62^, ^63^, ^64^, ^66^, ^67^, ^68^, ^69^, ^70^, ^71^, ^72^, ^73^, ^74^, ^75^, ^76^, ^78^, ^79^, ^80^, ^81^, ^82^, ^83^, ^84^	44^32^, ^33^, ^34^, ^35^, ^36^, ^37^, ^38^, ^39^, ^40^, ^41^, ^42^, ^43^, ^44^, ^45^, ^46^, ^47^, ^49^, ^50^, ^51^, ^52^, ^53^, ^54^, ^55^, ^56^, ^57^, ^58^, ^59^, ^60^, ^61^, ^62^, ^63^, ^64^, ^66^, ^67^, ^68^, ^69^, ^70^, ^71^, ^72^, ^73^, ^74^, ^75^, ^76^, ^77^ reviews that utilise BH viewpoints used synthesised evidence to understand if viewpoints were met, though not all explained how evidence was used or the criteria for a viewpoint to be met. There was a mix in synthesised evidence being narratively assessed only, statistically analysed only, or both narratively and statistically assessed to determine if viewpoints were met (see Section ‘Indicators used for meeting Bradford Hill viewpoints’ for more detail). Additional detail on how evidence was synthesised for all CAAs can be found in Section 3.2
Conclusion	53/53, 100%^32^, ^33^, ^34^, ^35^, ^36^, ^37^, ^38^, ^39^, ^40^, ^41^, ^42^, ^43^, ^44^, ^45^, ^46^, ^47^, ^48^, ^49^, ^50^, ^51^, ^52^, ^53^, ^54^, ^55^, ^56^, ^57^, ^58^, ^59^, ^60^, ^61^, ^62^, ^63^, ^64^, ^65^, ^66^, ^67^, ^68^, ^69^, ^70^, ^71^, ^72^, ^73^, ^74^, ^75^, ^76^, ^77^, ^78^, ^79^, ^80^, ^81^, ^82^, ^83^, ^84^	All reviews considered causality in their conclusions but it was unclear in three reviews whether a conclusion regarding a causal relationship was drawn.^77^, ^84^ For reviews utilising BH viewpoints, overall certainty of conclusion reflected overall certainty of viewpoints being met (see Section ‘Support for Bradford Hill viewpoints’). Twenty‐five reviews^32^, ^37^, ^38^, ^39^, ^40^, ^41^, ^42^, ^44^, ^45^, ^47^, ^48^, ^52^, ^53^, ^54^, ^56^, ^57^, ^58^, ^61^, ^65^, ^67^, ^76^, ^77^, ^82^, ^83^, ^84^ stated that they had found some evidence (with varying degrees of certainty) for a causal relationship between the exposure and outcome under study, while 18^33^, ^34^, ^43^, ^46^, ^49^, ^50^, ^51^, ^55^, ^59^, ^60^, ^62^, ^63^, ^64^, ^69^, ^70^, ^73^, ^74^, ^75^found limited or no evidence for a causal relationship. Nine studies said that they were not able to draw conclusive conclusions about a causal relationship and needed further evidence^35^, ^36^, ^66^, ^68^, ^71^, ^72^, ^78^, ^79^, ^80^, ^81^

There were seven SRs/RoRs^33^, ^39^, ^47^, ^49^, ^58^, ^71^, ^77^ (all utilising BH viewpoints) where causal assessment does not appear to have been incorporated into conduct of research objectives, review design, search strategies, inclusion criteria or data extraction. One SR^74^ appears to have incorporated CAA at each SR stage.

## 4 DISCUSSION

### 4.1 Overview of how CAAs are incorporated into population health SRs (objective 1 and 2)

Though there was some variation in how it was implemented, the most common CAA used by SRs/RoRs was BH viewpoints, which are considered among the most influential and comprehensive approaches to causal assessment.^88^, ^89^ Other CAAs included realist reviews and MRC guidance on natural experiments, which both have existing implementation guidance.^85^, ^86^ The remaining CAAs (horizontal SR; sign‐test hypothesis, and causal cascade model) were developed by SR authors, though the causal cascade model incorporated principles of DAGs. A common theme across the alternative CAAs was that most focused on one or two key aspects of causal assessment (e.g., one of the BH viewpoints). The overlap across CAAs also suggests that insight into implementing viewpoints should include reviews utilising BH viewpoints as well as reviews utilising alternative CAAs as both may offer useful insights for a given viewpoint. The comparison across CAAs suggests that while it may be preferable for some SRs/RoRs to take an in‐depth look at one characteristic of causal assessment, in another SR/RoR it would be preferable to consider many, depending on the focus and priorities for the review. Reviews that focus on one or two BH viewpoints (as opposed to several or all viewpoints) may find it easier to provide greater transparency about how the given viewpoint was implemented.

We found considerable variation in how BH viewpoints were used including their transparency, which was part of a broader understanding in how CAAs were implemented (objective 2). Transparent reporting of methods is a key component of SRs and lack of transparency in how CAAs were implemented in SRs/RoRs might result in assessments of causality not being reproducible which undermines the strength a SR/RoR. Based on our assessment of SR/RoRs using BH viewpoints, transparency of how viewpoints were implemented can be improved by (1) providing reasons for why certain viewpoints were used or omitted, (2) offering clear viewpoint definition and indicators, and (3) utilising a variety approaches for assessing support for viewpoints and applying viewpoints.

Firstly, as only three reviews explained why certain viewpoints were excluded,^42^, ^54^, ^57^ we are unsure if variation in which viewpoints were used reflected differences in viewpoints' perceived relevance for causal assessment or which viewpoints were more easily understood and applied. Moreover, only one‐third of reviews defined their included viewpoints while just 40% indicated how viewpoints were met. Limited clarity of why certain indicators were used makes it difficult to understand why there was, for example, a broad range for what was considered a ‘large’ effect estimate (strength of association) or what would be considered a ‘credible’ mechanism (plausibility). Finally, different approaches for assessing support for viewpoints and applying viewpoints improved overall transparency. Reviews that, for example, used both narrative and quantitative support for viewpoints provided more comprehensive assessment of the extent to which viewpoints were met than those that only provided quantitative or narrative assessments of support. Relatedly, reviews that implemented viewpoints across different study groupings (e.g., across all synthesised studies, across studies synthesised by exposure, and across individual studies) appear to more comprehensively consider causality than those that do not. Despite its importance, only a few reviews stood out as example of a rigorous and transparent application of BH viewpoints. Four reviews^38^, ^46^, ^54^, ^70^ defined the viewpoints, provided indicators and used both a narrative and quantitative rankings to describe certainty or likelihood of viewpoints having been met, with one of them explaining why certain viewpoints were not included.^54^

#### 4.1.1 Impact of incorporating CAAs on conducting SRs (objective 3)

Explicitly incorporating causal assessment into review objectives and CAAs into review study design, as most reviews did (see Table 4), are examples of how researchers can conduct causal SRs with clear research goals and explicit use of causal inference.^1^ To a lesser degree, CAA also impacted the search strategy, inclusion criteria and data extraction. It may be that so few reviews (3/53)^43^, ^74^, ^78^ designed their search to specifically identify terms such as ‘causal mechanism’ or ‘causality’ because doing so creates a low sensitivity search. It appears that an alternative approach is to have a highly sensitive search with a set of inclusion and exclusion criteria designed to identify studies most relevant to causal assessment, which about one quarter of reviews did (14/53, 26.4%). For example, the horizontal review and the MRC guidance on natural experiments review by Martin and colleagues designed their inclusion criteria to ensure their review included studies that assess bias and experimental evidence. Similarly, one quarter of reviews extracted information from included studies that supported causal assessment. Most CAAs were incorporated into the synthesis process. This includes using evidence to understand if BH viewpoints were met, synthesising evidence to understand causal mechanisms in realist reviews, sign test of the evidence for reverse causality, or test the evidence for statistical heterogeneity (causal cascade model). Finally, all reviews drew conclusions regarding causal relationships, suggesting it is a key component of a causal SR.

### 4.2 Strengths and weaknesses of methodological review

This methodological review is the first we have identified that summarises the use of causal approaches in SRs in current practice. It builds on literature exploring the use of SRs in causal assessment^11^, ^90^ and aiming to improve transparency and robustness around causal assessment.^1^, ^91^ Our findings are consistent with criticisms of causal SRs that there is no consensus on how to conduct a causal SR, though we found this variety may in fact strengthen causal assessment in SRs. The range of CAAs and variety in how a given CAA was implemented (both within BH viewpoints and across CAAs that utilised one or two BH viewpoints) provide many examples of causal SR that may be of use to different causal SRs with different areas of focus. In other words, it may be more relevant (given the exposure/outcome relationship under study, type of evidence available, main point of disagreement in the literature) for some reviews to focus on the BH viewpoint of experiment or temporality and for others to focus on several viewpoints in less detail.

This review has several limitations. The primary limitation in this methodological review was that the search was not sufficiently sensitive to identify reviews that did not use causal language in their title or abstract. Thus, we missed reviews that either implicitly applied causal approaches (such as sensitivity analyses for unmeasured confounding) or explicitly applied causal approaches but did not reference them in the title or abstract.^7^ We did not limit the search to specific population health topics, such as sexual health or men's health, as we aimed to include a broad range of population health SRs. That is, we designed the review to help us explore the range of possible CAAs across a broad area rather than exploring in greater detail issues of causal assessment specific to a particular topic. It is possible that we have overlooked useful insights from SRs of NRSs in subject areas outside population health. In addition, we focused our search on SRs/RoRs as they are considered the gold standard of evidence synthesis, so we may have missed additional CAA used by non‐SRs/RoRs. Relatedly, due to the limited number of reviews utilising alternative CAAs, we were only able to describe differences in how BH viewpoints were implemented. Moreover, we did not critically appraise the reviews and thus did not account for quality of SRs in our consideration of how causal approaches were applied.

## 5 IMPLICATIONS FOR FUTURE CONDUCT OF CAUSAL SRS

The range of CAAs, including variation across reviews that applied BH viewpoints, offer examples of how the same characteristic of causality could be implemented. Alternative CAAs that focus on one or two viewpoints appear to go into greater detail on those viewpoints (compared to reviews incorporating BH viewpoints) both in how the viewpoint is implemented and also appear to present greater transparency about how it has been implemented. However, reviews incorporating BH viewpoints (even though most did not use all nine viewpoints) appear to consider a broader range of characteristics of causality than the CAAs we identified. Investigators aiming to conduct causal SRs may need to consider which balance of depth and breadth is most appropriate for their consideration of a putative causal relationship.

Investigators should consider a range of CAAs and choose the approach that provides the greatest insight into whether a causal relationship exists, and this is especially true of BH viewpoints. This finding is consistent with an earlier theoretical comparison of BH viewpoints with other CAAs to elucidate viewpoints' theoretical underpinnings; our findings suggest that alternative CAA offer practical examples for improving the way individual viewpoints are implemented. For instance, the causal cascade model approach for evaluating heterogeneity and the horizontal review approach to evaluate the impact of different study designs the potential biases associated with each are potentially valuable for implementing the BH viewpoints consistency and strength of association. Formal testing (horizontal review, sign‐testing mechanisms) and comprehensive evaluation (realist review) of putative mechanisms is necessary to increase transparency around assumptions of plausibility. The MRC guidance on natural experiments lays out the analytical methods and study designs useful for implementing experiment. Falsification outcomes, as used by the MRC guidance on natural experiments, or comparing the associations of different exposure/outcome groupings, as the horizontal review and sign‐testing mechanism CAA did, may be useful approaches to evaluating specificity. Moreover, coherence and analogy were two of the three most infrequently used viewpoints. Though we are unsure why they were excluded, as they were used by fewer than half of reviews and as they were the only viewpoints that did not overlap with the alternative CAAs, their utility in causal assessment is not clearly supported.

SRs/RoRs applying BH viewpoints varied in how the viewpoints were implemented and transparency reporting on implementation. We found that transparent reporting of *why* viewpoints were implemented in a certain way (or considered not at all) is potentially as important as *how* viewpoints were implemented. That is, it may be more useful to understand why viewpoints have been excluded than to apply all nine viewpoints. Clarity, such as in defining viewpoints and providing criteria for how they may be met, also increases transparency reporting BH viewpoints. Where possible, we believe a more comprehensive approach to implementing the viewpoint is preferable. For example, reviews that describe the support for each viewpoint both narratively and quantitatively (e.g., strong/moderate/weak) offer greater transparency of how support for each viewpoint was considered. Transparent reporting of how viewpoints were implemented may clarify inconsistencies in how BH viewpoints were used.

## 6 CONCLUSION

This methodological review has evaluated how SRs/RoR that assess causality (‘causal reviews’) in population health research are conducted and reported. It contributes to the literature aimed at improving causal assessment in SRs, for which there are currently no established guidelines. While our goal was not to propose guidelines, our findings suggest overlap across the CAAs with BH viewpoints such that alternative CAAs appear to emphasise one or two viewpoints. This indicates that alternative CAAs should be used to inform, and improve, how BH viewpoints are implemented. Moreover, as there are also no guidelines for incorporating BH viewpoints, the most commonly applied CAA, we identified five key areas where reviews can be transparent: reasons for excluding viewpoints; viewpoint definition; viewpoint indicators; support for viewpoints; and application of viewpoints. The more transparent and clear reviews are about how CAAs are implemented, the greater clarity there is likely to be on how CAAs impact different SR stages which was not always clear. Overall, we found that clarity, transparency and engagement with other CAAs are the key approaches to conducting a causal SR.

## AUTHOR CONTRIBUTIONS

MS led (and AP, HT, and SVK supervised) conceptualization, methodology, investigation, analysis, and writing ‐ original draft. AP, HT, and SVK also validated findings and contributed to writing ‐ review & editing.

## FUNDING INFORMATION

Michal Shimonovich received funding from the Medical Research Council and the Medical, Veterinary and Life Sciences School at the University of Glasgow for her PhD. The author received no additional financial support for the research, authorship, and/or publication of this article.

## CONFLICT OF INTEREST

The authors declare there is no conflict of interest.

## Supporting information


**Appendix S1** Supporting Information.

## Data Availability

Data sharing is not applicable to this article as no new data were created or analysed in this study.
