# Supplementary material for: Causal assessment in evidence synthesis: A methodological review of reviews
Source: Res Synth Methods. 2022 Jun 9;13(4):405–23. doi: 10.1002/jrsm.1569 (PMC9543433; doi:10.1002/jrsm.1569)
Supplement: Supplementary file 1 — Appendix S1 Supporting Information. [file JRSM-13-405-s001.docx]

# Appendix A - Search strategy

Database(s): **Ovid MEDLINE(R) and In-Process & Other Non-Indexed Citations**1946 to February 04, 2020**, Embase**1996 to 2020 Week 05
Search Strategy:

| **#** | **Searches** | **Results** |
| --- | --- | --- |
| 1 | Meta-Analysis as Topic/ | 46035 |
| 2 | meta analy$.tw. | 366722 |
| 3 | metaanaly$.tw. | 11459 |
| 4 | Meta-Analysis/ | 286345 |
| 5 | (systematic adj (review$1 or overview$1)).tw. | 352512 |
| 6 | exp Review Literature as Topic/ or exp Review/ | 4880707 |
| 7 | data extraction.ab. | 43312 |
| 8 | selection criteria.ab. | 63872 |
| 9 | 1 or 2 or 3 or 4 or 5 or 6 or 7 or 8 | 5175676 |
| 10 | animal/ | 7453501 |
| 11 | human/ | 34412543 |
| 12 | 10 not (10 and 11) | 5277193 |
| 13 | 9 not 12 | 4997588 |
| 14 | (causal or causation or causal assessment or causality).ti. | 24988 |
| 15 | causality/ | 18687 |
| 16 | (directed acyclic graph* or causal pie* or Bradford Hill).ab,ti. | 1953 |
| 17 | GRADE.ab,ti. and 14 | 171 |
| 18 | 14 or 15 or 16 or 17 | 42814 |
| 19 | 13 and 18 | 7409 |
| 20 | 6 or 7 or 8 | 4919822 |
| 21 | systematic*.ab,ti. | 943914 |
| 22 | 20 and 21 | 364764 |
| 23 | 1 or 2 or 3 or 4 or 5 or 22 | 726207 |
| 24 | 18 and 23 | 1727 |
| 25 | remove duplicates from 24 | 1320 |

# Appendix B - Data extraction form

| **Study information** | | | | | | | | | |
| --- | --- | --- | --- | --- | --- | --- | --- | --- | --- |
| Author, year | Type of review | Study design of included studies | Population | Exposure | Outcome | Category of topic | Main causal assessment approach | Additional causal approaches | Critical appraisal tool |
| **Conduct of systematic review stages –evidence where causal assessment approach appeared to be incorporated into review stages** | | | | | | | | | |
| Author, year | Research Objective | Method for causal assessment | Inclusion/ exclusion criteria | Search terms | Data extraction | Further analysis in results /discussion | | Conclusion | |
| **Bradford Hill viewpoints – applied to each viewpoint. NA If viewpoint not used, NR if not reported** | | | | | | | | | |
| Author, year | Included in causal assessment (yes, no) | Viewpoint definition | Indicator for viewpoint being met | | Outcome for viewpoint | Ranking method used for certainty of viewpoint | | Application (across evidence, on each study, for exposure/outcome relationship, other) | |
| **Other approaches** | | | | | | | | | |
| Author, year | | Approach name | | Approach description | | Evidence of application | | Key features | |

# Appendix C – Study information

| Author, year | Included study design categories | Population of included studies | Exposure of included studies | Outcome | Category of topic by exposure | Main causal approach | Critical appraisal tool |
| --- | --- | --- | --- | --- | --- | --- | --- |
| Bass, 2004 (1) | All study designs | Adolescents (<14 years old) | Chronic or intermittent hypoxia | Congenital heart disease (CHD), sleep-disordered breathing (SDB), asthma, chronic ventilator impairment, and respiratory instability | Respiratory | Evidence based paediatrics and child health (EBPCH) (based on Bradford Hill) | US preventive services task force (USPSTF) |
| Biddle, 2016* (2) | Systematic review or meta-analyses and their included primary studies | Adults (> 18) | Sedentary behaviour | All-cause mortality | Occupational health | Bradford Hill | None |
| Biddle, 2017A* (3) | Systematic reviews | Adolescents (<19 years old) | Sedentary behaviour | Weight status or adiposity | Occupational health | Bradford Hill | AMSTAR |
| Biddle, 2017B* (4) | Systematic reviews | Adults (> 18 years old) | Sedentary behaviour | Weight status or adiposity | Occupational health | Bradford Hill | AMSTAR |
| Blair, 2014 (5) | Cohort studies | Neighbourhoods | Validated neighbourhood-level exposure variable | Depression or depressive symptoms | Environmental health | Realist review | Heller checklist for public health research and authors’ own tool |
| Boniface, 2017 (6) | Any study design | Adults | Interventions or policies leading to changes in the minimum price of alcohol | Alcohol sales, consumption, morbidity and mortality | Alcohol | Bradford Hill | Quantitative studies: Effective public health practice project's (EPHPP) tool  Qualitative studies and SRs: Critical appraisal skills programme (CASP) tools |
| Britton 2000 (7) | Case control or cohort studies | Not specified | Binge drinking | Cardiovascular disease | Alcohol | Bradford Hill | None |
| Bruce, 2013 (8) | Case reports; cross-sectional or cohort studies; randomised controlled trials | Children | Household air pollution (e.g., use of solid fuel for cooking) | Pneumonia, low birth weight, pre-term birth, stillbirth, stunting, and all-cause mortality | Environmental health | Bradford Hill | GRADE |
| Chen, 2017 (9) | Randomized controlled trials | Women at average-risk for breast cancer | Screen-detected breast cancer, interval cancers | Results of advanced breast cancer and breast cancer mortality after the follow-up of trials | Diagnostics | Causal cascade model | Jadad scoring system |
| Coffey, 2018 (10) | Interventional or observational studies (design not specified) | All humans | Socioeconomic or environmental variable | Group A Streptococcal  (GAS) infection (e.g., rheumatic heart disease (RHD) and from acute rheumatic fever (ARF)) | Inequalities | Bradford Hill | National institute of health study quality assessment tool |
| DeBono 2012 (11) | Cross-sectional or cohort studies; natural experiments | Adults (predominantly non-elderly adults) | Participation in the food stamp programme | Changes in body mass index (BMI) | Nutritional health | Realist Review | Checked for selection bias |
| Degelman, 2017 (12) | Case-control with comparator, cross-sectional or cohort studies | Adults | Active, passive, prenatal smoking | Clinically isolated syndrome (CIS) turning into clinically definite multiple sclerosis (CDMS); relapsing-remitting multiple sclerosis (RMS) turning into secondary-progressive multiple sclerosis (SPMS) | Smoking | Bradford Hill | GRADE |
| Fenton, 2011 (13) | in vitro animal experiments; prospective cohort studies; randomized controlled trials | Adults | Acid-base intake manipulated through supplemental salts (such as potassium bicarbonate) or through foods | Outcomes related to bone health or osteoporosis (bone strength as measured with biopsy, fractures, change of bone mass density, calcium balance, bone resorption markers, urine calcium) | Nutritional health | Bradford Hill | Cochrane risk of bias tool |
| Grant, 2013 (14) | Ecological studies (design not specified) | Adults | Exposure to e-waste and waste electrical and electronic equipment | Mental health and neurodevelopment, physical health, education, violence, and criminal behaviour | Environmental health | Bradford Hill | World cancer research fund (WCRF) tool |
| Hanioka, 2011 (15) | Cross-sectional or cohort studies | Adults | Exposure to smoking (current smoker, former smoker and non-smokers) | Tooth loss | Smoking | Bradford Hill | Newcastle–Ottawa scale |
| Hughes, 2014 (16) | Case reports; cross- sectional or cohort studies | All humans | Diisocyanates | Neurotoxic effect | Environmental health | Bradford Hill | None |
| Kerper, 2015 (17) | Cross-sectional or cohort studies | Adults | Pleural plaques | Lung function tests | Environmental health | Bradford Hill | None |
| Khan, 2020 (18) | Cross-sectional or cohort studies | Adults | Depression | Coronary atherosclerosis | Mental health | Bradford Hill | National institute of health quality assessment (NIH) scores |
| Kim, 2014 (19) | Case control, cross-sectional or cohort studies | Adults (>18 years old) | Brominated flame retardants | Loss of health (e.g., diabetes, neurobehavioral and developmental disorders, cancer, reproductive health effects and alteration in thyroid function) | Environmental health | Bradford Hill | World cancer research fund (WCRF) tool |
| Kroger, 2015 (20) | All study designs | Adults (from industrialized country, as defined by OECD membership status) | Socioeconomic status (e.g., education; occupation; income and wealth; labour market participation and success; excluded are among others studies on subjective social status, status of recipient of social benefits) | All health outcomes (e.g., physical health mental health; subjective health; sickness absence; biomarkers) | Inequalities | Sign-Test hypotheses | Low, medium, high quality (tool not specified) |
| Kuper, 2009 (21) | Genetic association studies; observational studies (design not specified), and randomized controlled trials | Adults (patients with existing coronary disease) | Depression, exercise, c reactive protein, and diabetes | Fatal coronary heart disease and non-fatal myocardial infarction (aetiological and prognostic studies) and, for prognostic studies only, all-cause mortality | Mental health | Horizontal Systematic Review | Scottish intercollegiate guideline network (SIGN) and fourth joint European societies tool |
| Lardon, 2014 (22) | Cross- sectional or cohort studies | Adolescents (<19 years old) | Puberty (explaining how the puberty score was obtained and using a measure, stated to be valid and reliable) | Back pain | Child's health | Bradford Hill | Newcastle–Ottawa scale |
| Leboeuf-yde 2000 (23) | Case control, cross-sectional or cohort studies | Adults | Body weight; body weight in relation to height skinfold thickness | Lower back pain | Nutritional health | Bradford Hill | None |
| Livesey, 2019a (24) | Prospective cohort studies | Adults | Glycaemic index (GI) and glycaemic load (GL) | Myocardial infarction (MI); fatal corollary heart disease (CHD) | Nutritional health | Bradford Hill | Newcastle–Ottawa scale |
| Livesey, 2019b (25) | Prospective cohort studies | Adults | Dietary glycaemic index  (GI) and glycaemic load (GL) | Type 2 diabetes (T2D) | Nutritional health | Bradford Hill | Newcastle–Ottawa scale |
| Martin, 2014 (26) | Cross-sectional or cohort studies using advanced analytical tools | Adults | Urban built environment characteristics | Obesity | Environmental health | Medical Research Council (MRC) Guidance on Natural Experiments | None |
| Mente, 2009 (27) | Prospective cohort studies; randomized controlled trials | Adults | Estimates of dietary intake using conventional dietary assessment tools (e.g., food frequency questionnaires, food records, or 24-hour diet recall) | Coronary heart disease (CHD), ischemic heart disease (IHD), and fatal or nonfatal myocardial infarction | Nutritional health | Bradford Hill | Low, medium, high risk of bias (tool not specified) |
| Micha, 2017* (28) | Systematic reviews or meta-analyses of prospective cohort studies or randomized controlled trials | Adults | Dietary factors (focused on foods, excluded overlapping component (e.g., included whole grains, fruits, and vegetables; and excluded dietary fibre)) | Cardiovascular disease (CVD), coronary heart disease (CHD), stroke, or diabetes | Nutritional health | Bradford Hill | World Cancer Research Fund/American Institute for Cancer Research (WCRF/AICR) criteria |
| Molenberg, 2019 (29) | All study designs measuring cycling before and after intervention | Adults (>16 years old) | Infrastructural intervention to promote cycling | Any measure of cycling as outcome | Environmental health | Medical Research Council (MRC) Guidance on Natural Experiments | Community Preventive Services Task Force (CPSTF) to assess study design and the Effective Public Health Practice Project (EPHPP) tool |
| Moore, 2017 (30) | Cross-sectional or cohort studies | Child or adolescent | Bullying victimization as a child or adolescent | Mental health problems, specifically depression, anxiety, self-harm, and suicidal behaviour | Mental health | Bradford Hill | World Cancer Research Fund (WCRF) |
| Norman, 2012 (31) | Case control, cross-sectional or cohort studies | Children | Non-sexual child maltreatment | Mental and physical health | Child's health | Bradford Hill | Newcastle–Ottawa scale |
| Norman, 2016 (32) | Cross-sectional or cohort studies,  randomized controlled trials; meta-analyses or reviews | Children | Food marketing | Children’s food behaviours, including food preferences and choices, short-term food consumption and usual dietary intake | Nutritional health | Bradford Hill | None |
| Reiss 2015 (33) | Cross-sectional or cohort studies | Humans (adults, children) | Exposure to organophosphorus (OP) insecticides | Birth outcomes and results of neurodevelopmental testing | Environmental health | Bradford Hill | Evaluated confounding and selection bias (tool not specified) |
| Roffey, et al 2010 (#60) (34) | Case control, cross-sectional or cohort studies | Adults | Awkward postures (i.e., any kneeling or squatting activities, awkward back positions, or working in uncomfortable postures) | Lower back pain (LBP) (any; chronic; subacute; severe; chronic; sick leave because of LBP) | Occupational health | Bradford Hill | Newcastle–Ottawa scale |
| Roffey et al 2010 (#61) (35) | Case control, cross-sectional or cohort studies | Adults | Not specified or not reported and harmful sitting | Lower back pain (LBP) or injury (any; severe) | Occupational health | Bradford Hill | Newcastle–Ottawa scale |
| Roffey et al 2010 (36) | Case control, cross-sectional or cohort studies | Adults | Pushing or pulling | Lower back pain (LBP) (any; mild or moderate; chronic, subacute, or recurrent; severe; seeking medical care; sick leave because of LBP) | Occupational health | Bradford Hill | Newcastle–Ottawa scale |
| Roffey et al 2010 (#70) (37) | Case control, cross-sectional or cohort studies | Adults | Standing (harmful; slippery or uneven surfaces; elevated surfaces; not report) or walking (not reported) | Lower back pain (LBP) (any; mild or moderate; chronic; severe; seeking medical care) | Occupational health | Bradford Hill | Newcastle–Ottawa scale |
| Roffey et al 2010 (#73)(38) | Case control, cross-sectional or cohort studies | Adults | Manual handling (e.g., laying brick and assembling scaffolding) or assisting patients (e.g., transferring a patient between wheelchair and commode or moving a patient around on a bed) | Lower back pain (LBP) (any; mild; chronic, subacute, or recurrent; severe; seeking medical care; sick leave because of LBP) | Occupational health | Bradford Hill | Newcastle–Ottawa scale |
| Rozanski, 2015 (39) | All study designs | Professional musicians | Intensive playing | a) anatomical manifestation of focal, task-specific dystonia within predefined instrument groups; b) conclusions regarding the prevalence of dystonia in musicians | Occupational health | Bradford Hill | Newcastle–Ottawa scale |
| Scott, 2018 (40) | Longitudinal case control or cohort studies | Participants with psychosis or schizophrenia | Presence of tobacco smoking | Psychosis or schizophrenia diagnosis | Smoking | Bradford Hill | Newcastle–Ottawa scale |
| Sharpe, 2013 (41) | Cross-sectional, prospective or experimental studies | All humans (children, adolescents and adults) | Fat talking topics ((a) self‐comparison to ideal eating and exercise habits; (b) fears of becoming overweight; (c) how eating and exercise habits compare to others; (d) evaluation of others' appearances, and (e) meal‐replacements and muscle‐building strategies) | Body dissatisfaction | Mental health | Bradford Hill | Risk of bias by Fowkes and Fulton 2011 |
| Shuper, 2010* (42) | Systematic reviews or meta-analyses | Adults | Alcohol consumption | HIV | Alcohol | Bradford Hill | None |
| Smith, 2019 (43) | Cross-sectional, cohort, or intervention studies | All (children, adolescents, adults, older adults) | Environmental exposure | Physical activity (e.g., walking, cycling, exercise, transport,  mobility, movement) | Environmental health | Bradford Hill | None |
| Stahl, 2012 (44) | All study designs | Workers | Any | Kienböck’s disease (osteonecrosis of the lunate) | Occupational health | Bradford Hill | Criteria of the Oxford Centre for Evidence-Based Medicine |
| Stahl, 2013 (45) | All study designs | Workers | Repetitive, forceful, and ergonomically stressful work | De quervain tenosynovitis | Occupational health | Bradford Hill | Strengthening the reporting of observational studies in Epidemiology (STROBE) |
| Swain, 2020 (46) | Systematic reviews or meta-analyses | Adults (≥ 18 years old) | Postural curvature, static posture (sitting and standing) or dynamic/occupational movements (e.g., bending, twisting, lifting) | Self-reported lower back pain (LBP) symptoms or LBP-specific outcomes (e.g., activity limitation, work absence, care-seeking, medication use) | Occupational health | Bradford Hill | AMSTAR |
| Tischer, 2011 (47) | Cross-sectional, case control, or cohort studies | Children (<15 years old) | Mould, measured airborne or dust-borne fungal genera, and measured specific biomarkers of mould species | Health outcomes to physician-diagnosed allergic diseases (e.g., asthma, allergic rhinitis or hay fever and eczema, wheezing, itchy, blocked or running nose without having a cold, itchy skin rash and allergic sensitisation to inhalant allergens) | Environmental health | Bradford Hill | None |
| van Amsterdam, 2018 (48) | Not specified (most are retrospective or cross-sectional studies) | Young people (15–25 years old) | Some measures of smoking (e.g., starting age, number of cigarettes per day, smoking trajectories) | Measures of ADHD or ADHD symptoms | Smoking | Bradford Hill | None |
| Wai, 2010a (49) | Cross-sectional, case control, or cohort studies | Workers | Occupational lifting | Lower back pain (LBP) or injury (any; mild or moderate; chronic; severe; seeking medical care; sick leave because of LBP) | Occupational health | Bradford hilil | Newcastle–Ottawa scale |
| Wai, 2010b (50) | Cross-sectional, case control, or cohort studies | Workers | Bending or twisting | Lower back pain (LBP) (any; mild or moderate; chronic or recurrent; subacute; severe; seeking medical care; sick leave because of LBP) | Occupational health | Bradford Hill | Newcastle–Ottawa scale |
| Wai 2010c (51) | Cross-sectional, case control, or cohort studies | Workers | Occupational carrying | Lower back pain (LBP) or injury (any; severe; sick leave because of LBP) | Occupational health | Bradford Hill | Newcastle–Ottawa scale |
| Yassi, 2013 (52) | All study designs | Nursing personnel | Performing nursing tasks | Non-specific back pain or back injury | Occupational health | Bradford Hill | Critical appraisal skills program (CASP) |
| Zhou, 2015 (53) | Prospective cohort studies | Adults | Coffee intake | Endometrial cancer | Nutritional health | Bradford Hill | Newcastle-Ottawa scale, GRADE |

1. Bass JL, Corwin M, Gozal D, Moore C, Nishida H, Parker S, et al. The effect of chronic or intermittent hypoxia on cognition in childhood: a review of the evidence. Pediatrics. 2004;114(3):805-16.

2. Biddle SJ, Bennie JA, Bauman AE, Chau JY, Dunstan D, Owen N, et al. Too much sitting and all-cause mortality: is there a causal link? BMC Public Health. 2016;16:635.

3. Biddle SJH, Garcia Bengoechea E, Wiesner G. Sedentary behaviour and adiposity in youth: A systematic review of reviews and analysis of causality. International Journal of Behavioral Nutrition and Physical Activity. 2017;14 (1) (no pagination)(43).

4. Biddle SJH, Bengoechea Garcia E, Pedisic Z, Bennie J, Vergeer I, Wiesner G. Screen Time, Other Sedentary Behaviours, and Obesity Risk in Adults: A Review of Reviews. Current Obesity Reports. 2017;6(2):134-47.

5. Blair A, Ross NA, Gariepy G, Schmitz N. How do neighborhoods affect depression outcomes? A realist review and a call for the examination of causal pathways. Social psychiatry and psychiatric epidemiology. 2014;49(6):873-87.

6. Boniface S, Scannell JW, Marlow S. Evidence for the effectiveness of minimum pricing of alcohol: A systematic review and assessment using the Bradford Hill criteria for causality. BMJ Open. 2017;7 (5) (no pagination)(e013497).

7. Britton A, McKee M. The relation between alcohol and cardiovascular disease in Eastern Europe: explaining the paradox. J Epidemiol Community Health. 2000;54(5):328-32.

8. Bruce NG, Dherani MK, Das JK, Balakrishnan K, Adair-Rohani H, Bhutta ZA, et al. Control of household air pollution for child survival: estimates for intervention impacts. BMC Public Health. 2013;13(Supplement 3):S8.

9. Chen THH, Yen AMF, Fann JCY, Gordon P, Chen SLS, Chiu SYH, et al. Clarifying the debate on population-based screening for breast cancer with mammography: A systematic review of randomized controlled trials on mammography with Bayesian meta-analysis and causal model. Medicine (United States). 2017;96 (3) (no pagination)(e5684).

10. Coffey PM, Ralph AP, Krause VL. The role of social determinants of health in the risk and prevention of group A streptococcal infection, acute rheumatic fever and rheumatic heart disease: A systematic review. PLoS Neglected Tropical Diseases. 2018;12 (6) (no pagination)(e0006577).

11. DeBono NL, Ross NA, Berrang-Ford L. Does the Food Stamp Program cause obesity? A realist review and a call for place-based research. Health Place. 2012;18(4):747-56.

12. Degelman ML, Herman KM. Smoking and multiple sclerosis: A systematic review and meta-analysis using the Bradford Hill criteria for causation. Multiple Sclerosis and Related Disorders. 2017;17:207-16.

13. Fenton TR, Tough SC, Lyon AW, Eliasziw M, Hanley DA. Causal assessment of dietary acid load and bone disease: A systematic review & meta-analysis applying Hill's epidemiologic criteria for causality. Nutrition Journal. 2011;10 (1) (no pagination)(41).

14. Grant K, Goldizen FC, Sly PD, Brune MN, Neira M, van den Berg M, et al. Health consequences of exposure to e-waste: A systematic review. The Lancet Global Health. 2013;1(6):e350-e61.

15. Hanioka T, Ojima M, Tanaka K, Matsuo K, Sato F, Tanaka H. Causal assessment of smoking and tooth loss: a systematic review of observational studies. BMC Public Health. 2011;11:221.

16. Hughes MA, Carson M, Collins MA, Jolly AT, Molenaar DM, Steffens W, et al. Does diisocyanate exposure result in neurotoxicity? Clinical Toxicology. 2014;52(4):242-57.

17. Kerper LE, Lynch HN, Zu K, Tao G, Utell MJ, Goodman JE. Systematic review of pleural plaques and lung function. Inhalation Toxicology. 2015;27(1):15-44.

18. Khan SA, Shahzad U, Zarak MS, Channa J, Khan I, Ghani MOA. Association of Depression with Subclinical Coronary Atherosclerosis: a Systematic Review. Journal of cardiovascular translational research. 2020;20.

19. Kim YR, Harden FA, Toms LML, Norman RE. Health consequences of exposure to brominated flame retardants: A systematic review. Chemosphere. 2014;106:1-19.

20. Kroger H, Pakpahan E, Hoffmann R. What causes health inequality? A systematic review on the relative importance of social causation and health selection. European journal of public health. 2015;25(6):951-60.

21. Kuper H, Nicholson A, Kivimaki M, Aitsi-Selmi A, Cavalleri G, Deanfield JE, et al. Evaluating the causal relevance of diverse risk markers: horizontal systematic review. BMJ (Clinical research ed). 2009;339:b4265.

22. Lardon A, Leboeuf-Yde C, Le Scanff C, Wedderkopp N. Is puberty a risk factor for back pain in the young? A systematic critical literature review. Chiropractic and Manual Therapies. 2014;22 (1) (no pagination)(27).

23. Leboeuf-Yde C. Body weight and low back pain: A systematic literature review of 56 journal articles reporting on 65 epidemiologic studies. Spine. 2000;25(2):226-37.

24. Livesey G, Livesey H. Coronary Heart Disease and Dietary Carbohydrate, Glycemic Index, and Glycemic Load: Dose-Response Meta-analyses of Prospective Cohort Studies. Mayo Clinic Proceedings: Innovations, Quality and Outcomes. 2019;3(1):52-69.

25. Livesey G, Taylor R, Livesey HF, Buyken AE, Jenkins DJA, Augustin LSA, et al. Dietary glycemic index and load and the risk of type 2 diabetes: Assessment of causal relations. Nutrients. 2019;11 (6) (no pagination)(1436).

26. Martin A, Ogilvie D, Suhrcke M. Evaluating causal relationships between urban built environment characteristics and obesity: a methodological review of observational studies. Int. 2014;11:142.

27. Mente A, De Koning L, Shannon HS, Anand SS. A systematic review of the evidence supporting a causal link between dietary factors and coronary heart disease. Archives of Internal Medicine. 2009;169(7):659-69.

28. Micha R, Shulkin ML, Penalvo JL, Khatibzadeh S, Singh GM, Rao M, et al. Etiologic effects and optimal intakes of foods and nutrients for risk of cardiovascular diseases and diabetes: Systematic reviews and meta-analyses from the nutrition and chronic diseases expert group (NutriCoDE). PLoS ONE. 2017;12 (4) (no pagination)(0175149).

29. Molenberg FJM, Panter J, Burdorf A, Van Lenthe FJ. A systematic review of the effect of infrastructural interventions to promote cycling: Strengthening causal inference from observational data. International Journal of Behavioral Nutrition and Physical Activity. 2019;16 (1) (no pagination)(93).

30. Moore SE, Norman RE, Suetani S, Thomas HJ, Sly PD, Scott JG. Consequences of bullying victimization in childhood and adolescence: A systematic review and meta-analysis. World j. 2017;7(1):60-76.

31. Norman RE, Byambaa M, De R, Butchart A, Scott J, Vos T. The Long-Term Health Consequences of Child Physical Abuse, Emotional Abuse, and Neglect: A Systematic Review and Meta-Analysis. PLoS Medicine. 2012;9 (11) (no pagination)(e1001349).

32. Norman J, Kelly B, Boyland E, McMahon AT. The Impact of Marketing and Advertising on Food Behaviours: Evaluating the Evidence for a Causal Relationship. Current Nutrition Reports. 2016;5(3):139-49.

33. Reiss R, Chang ET, Richardson RJ, Goodman M. A review of epidemiologic studies of low-level exposures to organophosphorus insecticides in non-occupational populations. Critical Reviews in Toxicology. 2015;45(7):531-641.

34. Roffey DM, Wai EK, Bishop P, Kwon BK, Dagenais S. Causal assessment of awkward occupational postures and low back pain: results of a systematic review. Spine Journal. 2010;10(1):89-99.

35. Roffey DM, Wai EK, Bishop P, Kwon BK, Dagenais S. Causal assessment of occupational sitting and low back pain: results of a systematic review. Spine Journal. 2010;10(3):252-61.

36. Roffey DM, Wai EK, Bishop P, Kwon BK, Dagenais S. Causal assessment of occupational pushing or pulling and low back pain: results of a systematic review. Spine Journal. 2010;10(6):544-53.

37. Roffey DM, Wai EK, Bishop P, Kwon BK, Dagenais S. Causal assessment of occupational standing or walking and low back pain: results of a systematic review. Spine Journal. 2010;10(3):262-72.

38. Roffey DM, Wai EK, Bishop P, Kwon BK, Dagenais S. Causal assessment of workplace manual handling or assisting patients and low back pain: results of a systematic review. Spine Journal. 2010;10(7):639-51.

39. Rozanski VE, Rehfuess E, Botzel K, Nowak D. Task-Specific Dystonia in Professional Musicians. A Systematic Review of the Importance of Intensive Playing as a Risk Factor. Deutsches Arzteblatt International. 2015;112(51-52):871-7.

40. Scott JG, Matuschka L, Niemela S, Miettunen J, Emmerson B, Mustonen A. Evidence of a Causal Relationship Between Smoking Tobacco and Schizophrenia Spectrum Disorders. Front Psychiatr. 2018;9:607.

41. Sharpe H, Naumann U, Treasure J, Schmidt U. Is fat talking a causal risk factor for body dissatisfaction? A systematic review and meta-analysis. International Journal of Eating Disorders. 2013;46(7):643-52.

42. Shuper PA, Neuman M, Kanteres F, Baliunas D, Joharchi N, Rehm J. Causal considerations on alcohol and HIV/AIDS - A systematic review. Alcohol and Alcoholism. 2010;45(2):159-66.

43. Smith L, Foley L, Panter J. Activity spaces in studies of the environment and physical activity: A review and synthesis of implications for causality. Health and Place. 2019;58 (no pagination)(102113).

44. Stahl S, Stahl AS, Meisner C, Rahmanian-Schwarz A, Schaller HE, Lotter O. A systematic review of the etiopathogenesis of Kienbock's disease and a critical appraisal of its recognition as an occupational disease related to hand-Arm vibration. BMC Musculoskeletal Disorders. 2012;13 (1) (no pagination)(225).

45. Stahl S, Vida D, Meisner C, Lotter O, Rothenberger J, Schaller HE, et al. Systematic review and meta-analysis on the work-related cause of de Quervain tenosynovitis: a critical appraisal of its recognition as an occupational disease. Plastic and reconstructive surgery. 2013;132(6):1479-91.

46. Swain CTV, Pan F, Owen PJ, Schmidt H, Belavy DL. No consensus on causality of spine postures or physical exposure and low back pain: A systematic review of systematic reviews. Journal of Biomechanics. 2020;102 (no pagination)(109312).

47. Tischer C, Chen CM, Heinrich J. Association between domestic mould and mould components, and asthma and allergy in children: A systematic review. European Respiratory Journal. 2011;38(4):812-24.

48. van Amsterdam J, van der Velde B, Schulte M, van den Brink W. Causal Factors of Increased Smoking in ADHD: A Systematic Review. Substance use & misuse. 2018;53(3):432-45.

49. Wai EK, Roffey DM, Bishop P, Kwon BK, Dagenais S. Causal assessment of occupational lifting and low back pain: results of a systematic review. Spine Journal. 2010;10(6):554-66.

50. Wai EK, Roffey DM, Bishop P, Kwon BK, Dagenais S. Causal assessment of occupational bending or twisting and low back pain: results of a systematic review. Spine Journal. 2010;10(1):76-88.

51. Wai EK, Roffey DM, Bishop P, Kwon BK, Dagenais S. Causal assessment of occupational carrying and low back pain: results of a systematic review. Spine Journal. 2010;10(7):628-38.

52. Yassi A, Lockhart K. Work-relatedness of low back pain in nursing personnel: a systematic review. International journal of occupational and environmental health. 2013;19(3):223-44.

53. Zhou Q, Luo ML, Li H, Li M, Zhou JG. Coffee consumption and risk of endometrial cancer: a dose-response meta-analysis of prospective cohort studies. Scientific reports. 2015;5:13410.
